# Cariprazine Use in Combination With a Mood Stabilizer in First Episode Mania

**DOI:** 10.3389/fpsyt.2022.828088

**Published:** 2022-05-11

**Authors:** Roberto Palacios-Garrán, Vicent Llorca-Bofí, Gara Arteaga-Henriquez, Enrique del Agua

**Affiliations:** ^1^Department of Psychiatry, Mental Health, and Addiction, GSS–Hospital Santa Maria, Lleida, Spain; ^2^Department of Psychiatry, Hospital Universitari Vall d'Hebron, Barcelona, Spain; ^3^Psychiatry Genetics Unit, Group of Psychiatry, Mental Health and Addictions, Vall d'Hebron Research Institute (VHIR), Universitat Autònoma de Barcelona, Barcelona, Spain; ^4^Biomedical Network Research Center on Mental Health (CIBERSAM), Madrid, Spain; ^5^Department of Psychiatry and Forensic Medicine, Universitat Autònoma de Barcelona, Barcelona, Spain

**Keywords:** first episode mania, cariprazine, bipolar disoder, acute mania, presicion medicine, treatment adherence

## Abstract

**Background:**

Cariprazine's efficacy and safety have been previously tested in adult patients with acute mania associated with bipolar I disorder, but there is no available data in FEM. The objective of this study is to assess the efficacy and safety of cariprazine in combination with a mood stabilizer in treating FEM as well as to evaluate patients' adherence to the treatment.

**Methods:**

FEM patients were recruited from the acute inpatient unit at Lleida University Hospital Santa Maria, between January and June 2021. Their symptoms were evaluated using the Young Mania Rating Scale (YMRS) and the Clinical Global Impressions–Severity (CGI-S) scale at admission and at discharge. Akathisia was assessed using the Barnes Akathisia Rating Scale. Patient adherence to medication treatment was assessed 30 days after discharge using the Morisky, Green and Levine Medication Adherence Scale. Socio-demographic and clinical information were further collected.

**Results:**

Eleven patients with FEM were involved, seven women and four men. Their mean age was 26.00+/-6.37 years. Mean hospitalization was 17.36+/−4.7 days. Cariprazine was combined with a mood stabilizer: lithium in seven patients and divalproex in four. Mean YMRS change from baseline was −24.55+/−7.5 and the mean CGI-S change from baseline was −2.55+/−0.82. Regarding adverse events, two (18.2%) patients presented with akathisia. At the 30-day treatment-adherence assessment, six (54.5%) patients were adherent and four (36.4%) had moderate adherence.

**Conclusion:**

In this sample, cariprazine in combination with mood stabilizers proved to be safe and effective in the treatment of FEM with more than half the patients being adherent to treatment. Therefore, cariprazine add-on is a good choice for promoting the long-term adherence of patients, thus minimizing the risk of relapse and improving prognosis.

## Introduction

Bipolar disorder (BD) is a chronic and disabling mental disorder, characterized by recurrent mood episodes of depression, mania, hypomania and mixed affective states with periods of full or partial remission (1). It is associated with high burden of disease and psychosocial dysfunction, affecting more than 1% of the general population (2). Mania is the most recognizable phase of the disorder, and its presence is key for diagnosis (2). Its characteristic symptoms include, among others, grandiosity, reduced need for sleep, distractibility, increased flight of ideas, impulsivity, and occasionally, it is further accompanied by psychotic symptoms (3). Mania has been associated with impaired psychosocial functioning and cognition (4), and patients sometimes require hospitalization in order to stabilize their psychopathological condition (5).

Given the recurring nature of the disorder, the emphasis of treatment is not only on the resolution of acute symptoms, but also on the assurance of long-term prophylaxis of mood episodes (6). Therefore, treatment by a multidisciplinary team is recommended, combining psychological and pharmacological treatment options (7). Regarding the treatment of manic episodes, the main objectives are the resolution of acute manic symptoms, behavioural and cognitive symptoms as well as psychotic symptoms, if present (6). Clinical guidelines for the pharmacological treatment of acute mania offer recommendations based on evidence, safety, and tolerability (8). One of the most recently published ones is *The 2020 Royal Australian and New Zealand College of Psychiatrists clinical practice guidelines for mood disorders* (6) which recommends oral monotherapy, if possible, with aripiprazole, asenapine, risperidone, quetiapine or cariprazine. If monotherapy is insufficient, second-generation antipsychotics can be combined with a mood-stabilizing agent: lithium or valproate (6). Lithium is considered to be the gold standard for the maintenance treatment of BD; however, its onset of action is slower than that of antipsychotics in the treatment of acute mania (9). Therefore, many clinicians combine lithium or other mood stabilizers with an atypical antipsychotic in order to treat the manic phase of BD. In fact, a combination therapy is recommended as first-line treatment option with greater efficacy than monotherapy with lithium or divalproex alone [Ogawa et al. (10); Pacchiarotti et al. (8)]. This latter treatment approach was applied for the purposes of the present study in first-episode mania (FEM) patients.

For FEM patients, medication adherence is an important aspect to consider, as it impacts on the efficacy of pharmacotherapy and therefore later disease-outcome (11). Thus, treatment should be initiated with cautious use of medications and slow titration, as early experiences of tolerability and side-effects prime later expectations and subsequent adherence, especially in FEM (12).

Cariprazine is a dopamine D2–D3 partial agonist with high affinity to D3 receptors. It is approved for the treatment of schizophrenia and the depressive and manic/mixed episodes associated with bipolar I disorder by the Food and Drug Administration, and it has shown efficacy as adjunctive treatment for major depressive disorder (13). It binds with high affinity to dopamine D2 and D3 receptors and to serotonin 5HT1A and 5HT2B receptors and with moderate affinity to serotonin 5HT2A receptors (14). A distinctive characteristic of cariprazine is that it has the highest affinity for D3 receptors among other antipsychotics; in fact, it is greater than that of dopamine itself (14). This makes cariprazine the only antipsychotic that can occupy the D3 receptors in the presence of dopamine in the living brain (15). Three short-term clinical trials have confirmed the efficacy of cariprazine over placebo (16–18), and a long-term (19) clinical trial confirmed the safety and tolerability of cariprazine in patients with bipolar I mania. The dose range in mania is 3–6 mg/day (20) with treatment-emergent affective switches reported with very low doses (21). Based on cariprazine's good tolerability and safety profile, it could not only treat mania effectively, but also improve treatment-adherence, therefore improving long-term outcomes of patients with FEM (22).

Although clinical trials are the gold standard of clinical research, they have some disadvantages, including that the data is not generalisable, as there are marked differences between patients involved in clinical trials and those seen in real-world settings (23). For instance, patients enrolled in trials are carefully screened using rigorous criteria, and comorbidities and adjunctive medications are highly controlled – all these aspects do not seem feasible in clinical practice (24). Therefore, it is important to supplement the knowledge gained from clinical trials with data gained from real-world evidence, such as electronic health and medical records, electronic devices and applications, case series or observational and naturalistic studies (25).

The objective of this study is to assess cariprazine's efficacy and safety in combination with mood stabilizers in treating FEM as well as patients' adherence to the treatment.

## Methods

This study is an observational study including patients over the age of 18 with a diagnosis of FEM where the medical decision was taken to initiate cariprazine treatment before the start of the study. Patients were recruited from the acute inpatient unit at Santa Maria University Hospital (Lleida, Spain) between January and June 2021. Diagnosis was based on the clinical assessment of the presentation at first inpatient hospitalization, following the DSM-5 A-D criteria for a manic episode (3).

Patients with a Young Mania Rating Scale (YMRS) (26) [Spanish version (27)] total score ≥ 18 at admission were included. Exclusion criteria included the presence of mixed symptoms, previous manic or psychotic episodes; mental intellectual disability; previous antipsychotic-use; and manic episode attributable to the physiological effects of substances or other medical conditions.

All patients were treated with cariprazine flexible doses (3–6 mg/day) in combination with a mood stabilizer: lithium (800–1,200 mg/day) or divalproex (1,000–1500 mg/day). Following the local recommendations for inpatients with severe mania (8), in addition to the antipsychotic treatment, a mood stabilizer was introduced in all cases. No specific timing of the start of the mood stabilizer is provided by the guidelines, but authors chose to start it on day 2 of cariprazine treatment, as it is the usual practice in their hospital. Treatment with both medications were maintained.

Socio-demographic and clinical information were collected. Patients were evaluated using YMRS and Clinical Global Impressions-Severity of Illness (CGI-S) scales at admission and discharge. Response (≥50% reduction in YMRS score at discharge) and remission (YMRS score ≤ 12 at discharge) were further assessed, using conventional cut-off criteria (28).

Based on previous safety studies of cariprazine (19, 29), the development of akathisia was measured using the Barnes Akathisia Rating Scale (BARS) (30). Further safety assessment included the evaluation of insomnia, headache and suicidality using a clinical interview conducted by the treating psychiatrist. Clinical laboratory tests conducted at baseline and discharge evaluated prolactin and metabolic (total cholesterol, LDL, HDL, triglycerides, and fasting glucose) changes, in line with common clinical practice for therapeutic monitoring (31).

Patient adherence to medication treatment was assessed 30 days after discharge using the Morisky Green Levine Medication Adherence Scale (MGLS) (32). Patients were categorized as: MGL = 0–1 representing low adherence, MGL = 2–3 representing moderate adherence, and MGL = 4 representing high adherence.

Descriptive statistics were calculated for the demographic and safety data. For evaluating the change on the YMRS and CGI-S measures, the related-samples Wilcoxon Signed Rank Test was conducted.

The study was carried out following the latest version of the Declaration of Helsinki, and the local ethics committee approved the study (CEIC-2341).

## Results

For a summary of patient characteristics, refer to Table 1. Seven women and four males with FEM were included in the study (*N* = 11) with a mean age of 26 +/−6.37 years. Mean hospitalization for the observed episode was 17.36 +/−4.7 days. Mean cariprazine dose was 4.64 mg +/−1.25 mg/day, administered once daily in the morning. Lithium carbonate was given to seven patients with a mean dose of 1085.71 +/−157.36, and divalproex sodium to four patients with a mean dose of 1,125 +/−250 mg/day. Regarding psychiatric comorbid conditions, one patient had attention-deficit hyperactivity disorder, one had post-traumatic stress disorder and four had substance use disorder.

**Table 1:** Summary of patient characteristics.

**Total patients,** *N*	11
**Male,** *n* (%)	4 (36.4)
**Female,** *n* (%)	7 (63.6)
**Age,** ***n*** (%)	
0–19 years	1 (9.1)
20–25 years	5 (45.5)
26–30 years	2 (18.2)
31–36 years	3 (27.3)
**Duration of hospitalization,** ***n*** (%)	
0–9 days	0 (0)
10–15 days	4 (36.4)
16–20 days	3 (27.3)
21–25 days	4 (36.4)
Cariprazine dose^*^, *n* (%)	
3.0 mg/day	3 (27.3)
4.5 mg/day	4 (36.4)
6.0 mg/day	4 (36.4)
**Concomitant mood stabilizers and their dose,** ***n*** (%)	
*Lithium*	*7 (63.6)*
−800 mg/day	1 (9.1)
−1,000 mg/day	2 (18.2)
−1,200 mg/day	4 (36.4)
*Divalproex*	*4 (36.4)*
−1,000 mg/day	3 (27.3)
−1,500 mg/day	1 (9.1)
**Psychiatric comorbid conditions,** ***n*** (%)	
PTSD	1 (9.1)
ADHD	1 (9.1)
SUD	4 (36.4)
**DSM-5 criteria,** ***n*** (%)	
Bipolar I disorder, current or most recent episode manic with psychotic features	8 (72.7)
Bipolar I disorder, current or most recent episode manic without psychotic features	3 (27.3)

*PTSD, post-traumatic stress disorder; ADHD, attention-deficit/hyperactivity disorder; SUD, substance use disorder*.

* *Dose at discharge*.

**Figure 1:**
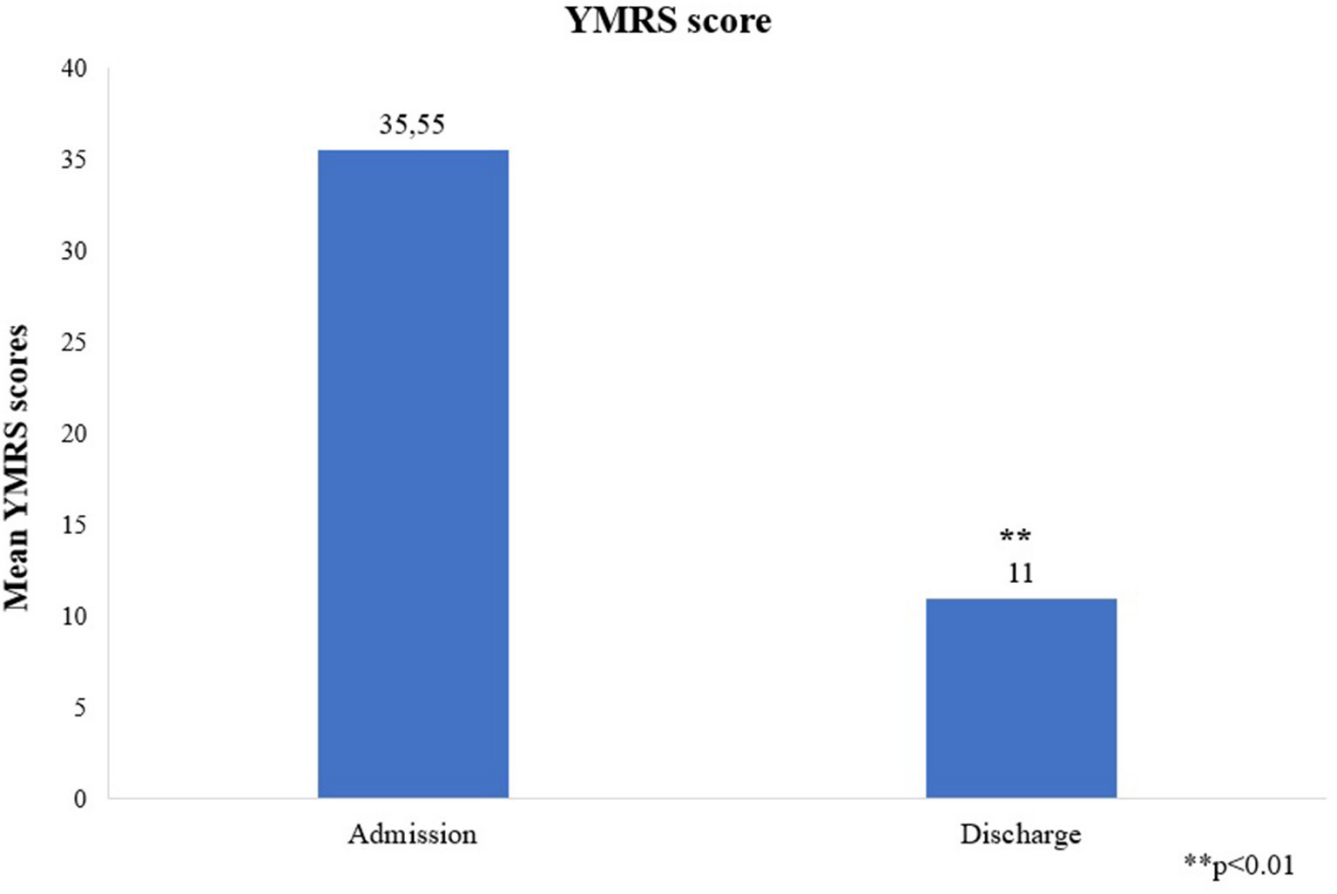
Mean YMRS scores at admission and discharge. YMRS, Young Mania Rating Scale. **p < 0.01.

**Figure 2:**
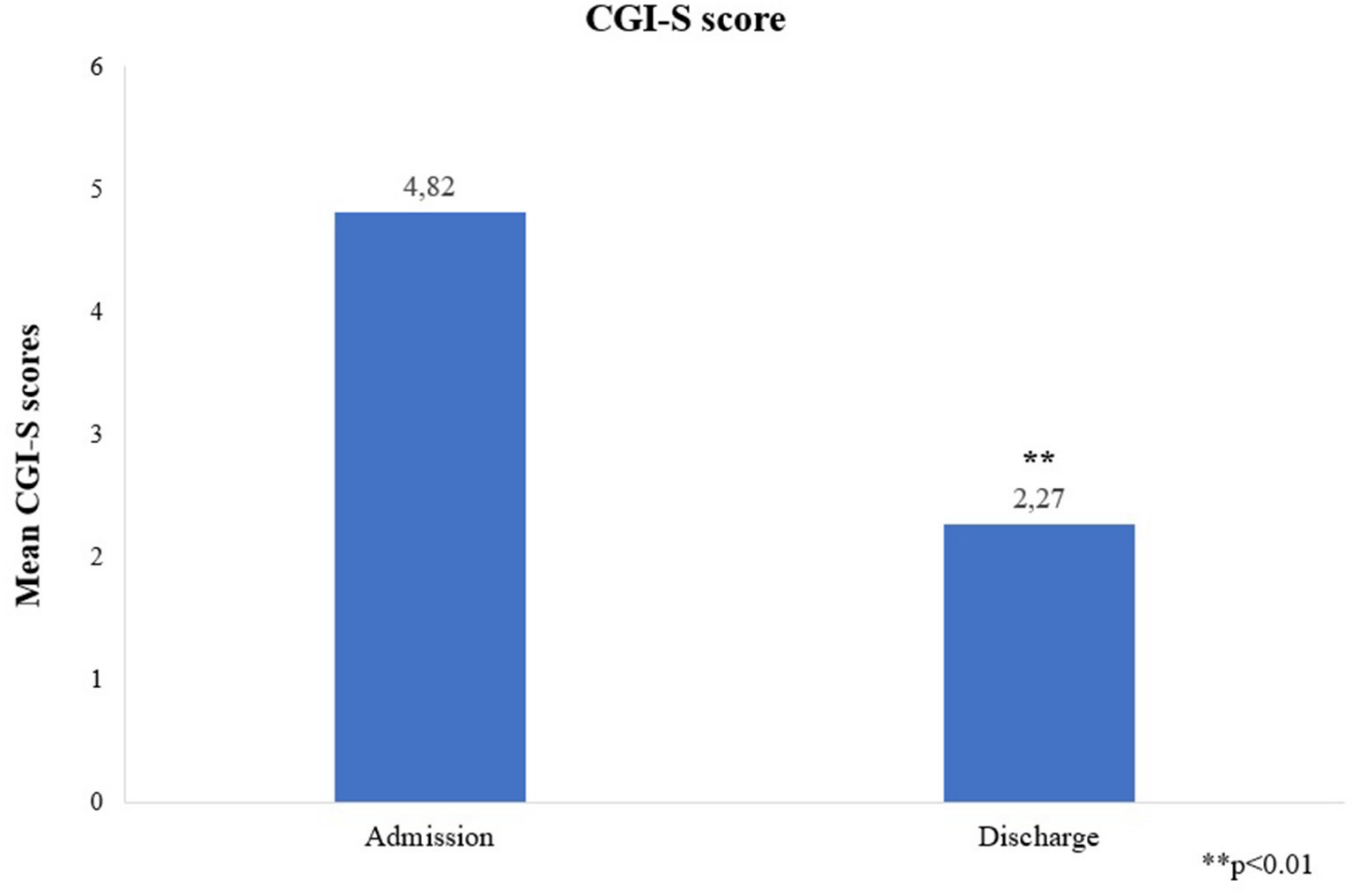
Mean CGI-S scores at admission and discharge. CGI-S, Clinical Global Impression – Severity Scale. **p < 0.01.

The mean YMRS score was 35.55 +/−7.79 at admission and 11 +/−2.19 at discharge, *p* = 0.003, change from baseline was −24.55 +/−7.5 (Figure 1). All patients achieved a clinically significant response (≥50% reduction in YMRS score at discharge). Eight (72.7%) patients achieved clinically significant remission (YMRS ≤ 12) and three (27.3%) patients showed minimal symptoms at the end of the hospitalization (YMRS = 13–19). Those with minimal symptoms at discharge showed psychotic symptoms at admission; had larger duration of untreated mania; and received the highest dose of cariprazine (6 mg/day) during the hospitalization. Mean CGI-S decreased from 4.82 +/−0.87 at admission to 2.27 +/−0.65 at discharge, *p* = 0.003, change from baseline is therefore −2.55 +/−0.82 (Figure 2).

For a summary of the safety outcomes, refer to Table 2. Regarding adverse events, two (18.2%) patients developed akathisia (one moderate and one marked), one (9.1%) experienced insomnia and two (18.2%) reported headache. No suicidal behaviour was noted in any of the patients. Mean change in prolactin level from baseline to discharge was −4.97 +/−5.05 ng/mL for females and −3.28 +/−2.7 ng/mL for males. Mean change of metabolic parameters was: 1.90 +/- 8.87 mg/dL for total cholesterol; 1.84 +/−13.49 mg/dL for LDL cholesterol; −2.97 +/−6.06 mg/dL for HDL cholesterol; 16.71 +/−21.09 mg/dL for Triglycerides; and 7.85 +/−13.78 for fasting glucose.

**Table 2:** Summary of the safety outcome measures.

**TE adverse events, *n* (%)**	
Akathisia	2 (18.2)
Headache	2 (18.2)
Insomnia	1 (9.1)
Suicidality	0 (0)
**TE laboratory changes, mean (SD)**	
Prolactin – males (ng/mL)	−3.28 (2.7)
Prolactin – females (ng/mL)	−4.97 (5.05)
Total cholesterol (mg/dL)	1.9 (8.87)
LDL cholesterol (mg/dL)	1.84 (13.49)
HDL cholesterol (mg/dL)	−2.97 (6.06)
Triglycerides (mg/dL)	16.71 (21.09)
Fasting glucose (mg/dL)	7.85 (13.78)
Adherence^*^, *n* (%)	
Low	1 (9.1)
Moderate	4 (36.4)
High	6 (54.5)

*TE, treatment-emergent*.

* *Based on the Morisky Green Levine Medication Adherence Scale*.

Regarding treatment-adherence, six (54.5%) patients displayed high adherence, four (36.4%) moderate adherence and one (9.1%) patient had low adherence to the medication, as shown by the MGLS score at day 30 after discharge.

## Discussion

This was the first study to specifically investigate cariprazine's efficacy in combination with a mood stabilizer in FEM. In this sample, mean YMRS scores and CGI-S scores showed a great reduction from admission to discharge with all patients achieving clinically significant response. Furthermore, 72% patients achieved clinically significant remission.

These findings are in line with those of clinical trials. The short-term efficacy and safety/tolerability of cariprazine was confirmed in three 3-week placebo-controlled studies (16–18) in adult patients with acute manic or mixed episodes associated with bipolar I disorder. Flexible-dose cariprazine 3–12 mg/day was used in two studies (16, 17) and a fixed/flexible dose scheme (3–6 mg/day or 6–12 mg/day) was used in the third (18). In each trial, improvement from baseline to week 3 in YMRS score, CGI-S scores as well as rates of response were significantly greater for cariprazine- than for placebo-treated patients. Remission rates also showed statistical significance in favour of cariprazine over placebo (33). This aspect is of high significance, as the persistence of symptoms after the acute treatment of mania was shown to be associated with worse illness-outcome and an increased risk of relapse (33). Therefore, the fact that cariprazine patients achieved response and remission has clinical significance in the improvement of prognosis (33).

In general, the first stages of most diseases require a simpler approach and treatment response is usually more favourable, obtaining a greater benefit with less risk (34). After the first episode, multiple relapses and a progressive worsening of psychosocial functioning and cognition often occur. However, it is thought that first episodes represent a window of intervention to improve clinical results and patient's quality of life (5). Despite that, guidelines focusing on the treatment of FEM are scarce (35). Treatment in FEM yields complete remission of the manic syndrome in most cases, but it may take longer for males, younger patients, or those with psychotic features or a longer duration of untreated mania (11).

Furthermore, BD is associated with more frequent relapses than other psychiatric diseases (36) with non-adherence to pharmacological treatment (37) and residual symptoms after an acute manic episode being the best predictors of relapse (33). There are several factors influencing adherence: individual-specific sociodemographic factors, insight, cognition as well as illness-specific factors, like illness-severity or comorbidities (38). Of note, there are medication-specific factors as well, like the complexity of the medication regimen and side-effects (39). Regarding cariprazine, it is given orally once daily; can be taken with or without food; can be taken at any time of the day; and neither age, gender nor smoking influence dose administration (40), making the medication regimen easy to comply with.

Regarding side-effects, akathisia incidence in our sample (18.2%) was similar to those reported in acute double-blind studies (pooled data of the three short-term studies: 19.8% for the 3–6 mg/day dose-range) (29). Prevalence of insomnia and headache, (9.1 and 18.2%, respectively) were also similar to the outcomes of the pooled analysis (8.7, 13.7% in the same dose-range) (29).

Regarding the metabolic parameters, there was a slight increase observed in triglycerides, fasting glucose and cholesterol levels (except for HDL cholesterol) in our sample. These findings are generally similar to those observed in clinical trials (29). In addition, mean metabolic variations were inferior to 5% for total cholesterol and LDL cholesterol but not for HDL cholesterol; mean triglyceride variations were inferior to 20–30% and mean fasting glucose increase was inferior to 10 mg/dL, which are within normal ranges (31, 41).

Mean change in prolactin level from baseline to discharge in this sample was similar to those reported in *post-hoc* analyses conducted on pooled data of the three short-term studies Patel et al. (42). Decrease in prolactin level was seen as a consequence of the D2 partial agonism, especially in females.

Psychotic features are common in bipolar mania, some studies estimating it to be around 68% (43) which is similar to our sample, where 72.7% of the patients experienced psychotic symptoms. The presence of psychotic symptoms leads to an earlier age of onset and more severe mood episodes, requiring more frequent hospitalizations – making it crucial to find an effective treatment for this patient population (44). A study explored the pharmacological treatments and found that having bipolar mania with psychotic features is associated with receiving a combination therapy of an antipsychotic and an anticonvulsant agent (44). However, there is no evidence of superiority of any first-line antipsychotic (8). In our study, cariprazine in combination with lithium or divalproex sufficiently addressed psychotic symptoms in FEM.

Overall, cariprazine has an easy medication regimen and a favourable safety profile. Cariprazine's long-term tolerability was demonstrated by Ketter and colleagues (19) in a 16-week open-label cariprazine 3–12 mg/day study, with akathisia being a common adverse event. They showed low rates of sedation or weight gain and although akathisia occurred in one-third of the patients, it yielded low rates of discontinuation as it was managed effectively. Therefore, cariprazine could be a good choice of pharmacotherapy to ensure adherence from the first stages of the disease. In terms of 30-day adherence, in our study, six (54.5%) patients displayed high adherence, four (36.4%) moderate adherence and one (9.1%) patient had low adherence to the medication. This is an encouraging data for adherence, but studies are needed to examine this further.

One of the limitations of the data presented here is the small sample size. In addition, conclusions regarding the efficacy and risk/benefit profile of cariprazine are difficult to be drawn on due to the lack of an active comparator; multiple doses; and the concomitant medication. Also, many of the patients in this sample had a psychiatric comorbid condition which is known to negatively influence many aspects of the disorder – including less favourable treatment response, especially to lithium – making it complicated to draw accurate conclusions from these findings. Furthermore, patients were followed up for a short period only, warranting the need for longer observations to conclude long-term effects.

## Conclusions

In this sample, cariprazine in combination with a mood stabilizer (lithium or divalproex) was effective in resolving the acute manic episode of FEM patients and it proved to be safe and well-tolerated with a low rate of adverse effects. Since the BD internationals guidelines recommend choosing treatment based on not only efficacy, but also short-term and long-term safety and tolerability, cariprazine is a good choice of pharmacotherapy. Given cariprazine's gentle safety profile and ease of administration, it likely improves patient's adherence to treatment and therefore helps minimizing the risk of relapse and improves prognosis.

## Data Availability Statement

The raw data supporting the conclusions of this article will be made available by the authors, without undue reservation.

## Ethics Statement

The studies involving human participants were reviewed and approved by Comitè d'Ètica d'Investigació amb medicaments de Lleida (CEIC-2341). The patients/participants provided their written informed consent to participate in this study.

## Author Contributions

RP-G and VL-B were responsible for conception and design as well as initial drafting of the manuscript. GA-H and EA were responsible for revising the manuscript critically for important intellectual content of the version of the manuscript to be published. All authors read and approved the final manuscript.

## Funding

Gedeon Richter provided funds for the open access publication fees.

## Conflict of Interest

The authors declare that the research was conducted in the absence of any commercial or financial relationships that could be construed as a potential conflict of interest.

## Publisher's Note

All claims expressed in this article are solely those of the authors and do not necessarily represent those of their affiliated organizations, or those of the publisher, the editors and the reviewers. Any product that may be evaluated in this article, or claim that may be made by its manufacturer, is not guaranteed or endorsed by the publisher.
